# High Prevalence of Obesity Among Inner-City Adolescent Boys in the Bronx, New York: Forgetting Our Boys

**Published:** 2010-12-15

**Authors:** Carmen R. Isasi, Amy Whiffen, Yolanda Florez, Katherine Freeman, Judith Wylie-Rosett, Eleanor Campbell

**Affiliations:** Department of Epidemiology and Population Health, Albert Einstein College of Medicine of Yeshiva University; Albert Einstein College of Medicine of Yeshiva University, Bronx, New York; Albert Einstein College of Medicine of Yeshiva University, Bronx, New York; Albert Einstein College of Medicine of Yeshiva University, Bronx, New York; Albert Einstein College of Medicine of Yeshiva University, Bronx, New York; Lehman College, City University of New York, Bronx, New York

## Abstract

We examined sex differences in overweight and obesity in a sample of 1,619 inner-city adolescents. Participants were enrolled from 11 public schools in the Bronx, New York. The prevalence of overweight and obesity was 21.7% and 22.5%, respectively; prevalence of obesity was significantly higher among adolescent boys than adolescent girls (24.9 vs 20.1%). Childhood obesity is a public health concern in the United States, and the higher prevalence of obesity in adolescent boys requires additional attention.

## Objective

Childhood obesity is a growing concern in the United States. Data from the third National Health and Nutrition Examination Survey show that excess weight is associated with metabolic abnormalities such as dyslipidemia and insulin resistance (1). National surveys have reported a prevalence of overweight and of obesity of 34% and 18%, respectively, among youth aged 12 to 19 years (2). Low-income and minority youth, particularly Hispanics and African Americans, are the most affected (2-5).

Findings from recent studies challenge the common assumption that girls are at higher risk of overweight and obesity than boys (5,6). Among Mexican American adolescents, boys also have a higher prevalence of obesity than girls (6). A recent analysis of data from the National Longitudinal Study of Adolescent Health (Add Health), a national representative sample of adolescents in grades 7 through 12, found that during early adolescence, boys have a higher body mass index (BMI) than girls, although girls have a faster increase in BMI over the years (7). This disparity appears to persist as teenagers get older, but by age 20 the prevalence of obesity in adolescent girls gets closer to or higher than that of boys (6,7). Despite this reversal, a high prevalence of obesity among adolescent boys is still of concern. The Add Health study showed that 88% of adolescent boys remained obese as young adults (8).

The purpose of our study was to examine sex differences in overweight and obesity among inner-city adolescents in the Bronx, New York. Factors associated with excess weight may vary by sex, and treatment approaches may need to take into account these differences.

## Methods

### Study sample

This cross-sectional study took place during February through June and October through December of 2008. Inclusion criteria for this study included ability to speak and comprehend English and not being enrolled in special education classes. The Bronx has 350 public schools that enroll students in grades 7 through 10 (9). The 11 participating schools served 11,789 students in grades kindergarten through 12, and 2,707 students were in grades 7 through 10. We enrolled 1,809 adolescents in grades 7 through 10 from 11 public schools in the Bronx; 1,619 (90%) students provided height and weight data.

Mean age of the participants was 13.9 years (standard deviation, 1.4; range, 11-18 y), and 51% were female. The sample was 75% Hispanic, 6% non-Hispanic African American, and 19% other race/ethnicity; 79% of participants were born in the United States. Data on maternal education indicated that 23% of mothers did not graduate from high school, 23% were high school graduates, and 54% had some college education or more. Thirty-seven percent of adolescents lived with both biological mother and father.

### Procedures

The study was approved by the institutional review boards of the Albert Einstein College of Medicine and the New York City Department of Education. Before the start of data collection, letters that described the research study were mailed to parents. Parents were told to mail back a self-addressed, prepaid postcard or to notify a designated school official if they did not want their child to participate in the study. Before data collection, students had the choice to refuse participation or to sign an assent form if they agreed to participate. The participation rate was 68% (1,809 of 2,661). Reasons for nonparticipation were parental or student refusals (1% and 9%, respectively), letters to parents returned by the post office (2%), and absenteeism (20%).

Trained research staff measured students' height and weight by using a standardized procedure (10). Weight was taken on a Seca Robusta 813 digital scale (Seca GmbH & Co, Hamburg, Germany) while participants were wearing light clothing and no shoes. Standing height was measured with a Seca portable stadiometer 214 (Seca GmbH & Co, Hamburg, Germany) with a vertical backboard and a moveable headboard. These measures were obtained during physical education class, and privacy screens were used.

BMI and BMI percentiles were derived according to age- and sex-specific growth charts from the Centers for Disease Control and Prevention (11). Adolescents were classified as overweight (≥85th and <95th percentile), obese (≥95th percentile), or severely obese (≥99th percentile). Twelve adolescents were underweight (<5th percentile) and were excluded from the sample because the number was too low for meaningful comparisons. Because height and weight were not obtained on the day of consent, this information was missing for 190 students who were not present when research staff came back for these measures. Demographic characteristics were similar in the groups with and without height and weight data. Statistical analyses included calculation of percentage and mean differences between groups, which were tested using χ^2^ and *t* tests, respectively. Analyses were conducted using Stata statistical software release 10 (StataCorp LP, College Station, Texas).

## Results

Prevalence of overweight was 21.7%, and prevalence of obesity was 22.5%. These rates did not vary by age, grade, maternal education, or family structure. More Hispanic adolescents were obese than non-Hispanic adolescents (24% vs 17%, χ^2^ test, *P* = .003). Among non-Hispanic African American adolescents, prevalence of overweight was 16%, and prevalence of obesity was 19%. The prevalence of obesity was higher among adolescents born in the United States than foreign-born adolescents (24% vs 17%, χ^2^ test,* P* = .008), but rates of overweight were not statistically different (21% vs 24%, χ^2^ test, *P* = .21).

Prevalence of overweight was higher among adolescent girls than boys, but prevalence of obesity and severe obesity was higher among adolescent boys than adolescent girls (Table). These sex differences were significant for Hispanic adolescents but not for non-Hispanic African American adolescents. Among obese adolescents, 37% of boys and 21% of girls had a BMI at the 99th percentile or higher.

## Discussion

Findings from this inner-city and predominantly Latino sample of adolescents indicate that adolescent boys are at a higher risk of obesity than adolescent girls. Among the obese teenagers, a large proportion were found to be at a BMI percentile of 99th or higher — severely obese. Consistent with findings from a previous study, we found that US-born adolescents were more likely to be obese than foreign-born adolescents (12). Our results showed that 44% of adolescents had a BMI at the 85th percentile or higher for their given age and sex. This prevalence of excess weight is larger than that reported for public school students in New York City (39%) (13) and the South Bronx (38%) (14) and higher than the national estimates for children aged 12 to 19 years (2). These studies also observed similar sex differences, but, for the most part, did not identify these differences as an emergent public health problem.

Data from the National Health and Nutrition Examination Survey showed that, except for non-Hispanic African American adolescents, boys are more obese than girls, although the sex difference is smaller than in our study (2). In New York City, data from kindergarten to eighth-grade students showed that adolescent boys are more likely to be obese than adolescent girls in all ethnic/racial groups except for non-Hispanic African Americans (13). National estimates of severe obesity in children also show sex differences; boys have a higher prevalence (15). However, this prevalence is lower than the prevalence we observed. Reasons for this larger difference remain to be examined. The Bronx is the poorest borough in New York City, and its residents are at higher risk of having poor health (16). A large proportion of adolescents do not meet the recommendations for fruit and vegetable consumption (80%) and exercise (43%) (14). These characteristics, together with a larger percentage of Latino residents (17), who are the most affected by the obesity epidemic, may help explain the large sex disparities in overweight and obesity observed in our study.

Our study has several limitations. The cross-sectional design prevented us from examining whether the sex differences in overweight and obesity persist or reverse as teenagers get older. The participating schools were not randomly selected, so the study sample may not be representative of the adolescent population in the Bronx. Furthermore, the large percentage of absentees (20%), although it is consistent with absentee rates for the area (18), limits the generalizability of the study findings.

Long-term prospective studies showed that obesity in youth continues into young adulthood (8,19), and persistent obesity is associated with increased risk of diabetes and hypertension in young adults (20). Therefore, although sex differences in obesity appear to be reversed in young adulthood (6,7) and in middle age (21), obesity among adolescent boys is of concern, given the health consequences of excess weight in adulthood. Furthermore, little is known about the factors that put boys at a higher risk of obesity (4). This scarcity of research could contribute to a bigger public health problem for adolescent boys, especially among boys of Hispanic heritage. Whether preventive and treatment strategies should be tailored to adolescent boys, and how they should be tailored, needs immediate attention to ensure that boys become healthy adults.

## Figures and Tables

**Table:** Prevalence of Overweight and Obesity, by Sex, Among Inner-City Adolescents, Bronx, New York, 2008

Body Mass Index Category (Percentile)^a^	Total, % (N = 1,607)^b^	Girls, % (N = 816)	Boys, % (N = 791)	*P* Value^c^
Overweight (≥85th to <95th)	21.7	24.5	18.8	.006
Obese (≥95th)	22.5	20.1	24.9	.02
Severely obese (≥99th)	5.1	3.6	6.7	.004

a Body mass index percentiles were derived according to age- and sex-specific growth charts from the Centers for Disease Control and Prevention (11).

b Of the 1,619 students who participated, 12 were classified as underweight and were excluded from analysis.

c *P* values derived from χ^2^ analysis.
